# Health-related quality of life and a cost-utility simulation of adults in the UK with osteogenesis imperfecta, X-linked hypophosphatemia and fibrous dysplasia

**DOI:** 10.1186/s13023-016-0538-4

**Published:** 2016-11-28

**Authors:** Lydia Forestier-Zhang, Laura Watts, Alison Turner, Harriet Teare, Jane Kaye, Joe Barrett, Cyrus Cooper, Richard Eastell, Paul Wordsworth, Muhammad K. Javaid, Rafael Pinedo-Villanueva

**Affiliations:** 1 Nuffield Department of Orthopaedics, Rheumatology and Musculoskeletal Sciences, Oxford NIHR Musculoskeletal Biomedical Research Unit, Rheumatology and Musculoskeletal Sciences, University of Oxford, Oxford, UK; 2 Department of Public Health, University of Oxford, Oxford, UK; 3 MRC Lifecourse Epidemiology Unit, University of Southampton, Southampton, UK; 4 Academic Unit of Bone Metabolism, Metabolic Bone Centre, Northern General Hospital, Sheffield, UK; 5 The Botnar Research Centre, NIHR Oxford Musculoskeletal BRU, NDORMS, University of Oxford, Oxford, OX3 7HE UK

**Keywords:** Quality of life, Economic evaluation, Osteogenesis imperfecta, Fibrous dysplasia, McCune Albright syndrome, X-linked hypophosphatemia

## Abstract

**Background:**

Health-related quality of life of adults with osteogenesis imperfecta (OI), fibrous dysplasia (FD) and X-linked hypophosphatemia (XLH) remains poorly described. The aim of this study was to describe the HRQoL of adults with osteogenesis imperfecta, fibrous dysplasia and X-linked hypophophataemia and perform a cost-utility simulation to calculate the maximum cost that a health care system would be willing to pay for a hypothetical treatment of a rare bone disease.

**Results:**

Participants completed the EQ-5D-5 L questionnaire between September 2014 and March 2016. For the economic simulation, we considered a hypothetical treatment that would be applied to OI participants in the lower tertile of the health utility score.

A total of 109 study participants fully completed the EQ-5D-5 L questionnaire (response rate 63%). Pain/discomfort was the most problematic domain for participants with all three diseases (FD 31%, XLH 25%, OI 16%).

The economic simulation identified an expected treatment impact of +2.5 QALYs gained per person during the 10-year period, which led to a willing to pay of £14,355 annually for a health care system willing to pay up to £50,000 for each additional QALY gained by an intervention.

**Conclusions:**

This is the first study to quantitatively measure and compare the HRQoL of adults with OI, FD and XLH and the first to use such data to conduct an economic simulation leading to healthcare system willingness-to-pay estimates for treatment of musculoskeletal rare diseases at various cost-effectiveness thresholds.

**Electronic supplementary material:**

The online version of this article (doi:10.1186/s13023-016-0538-4) contains supplementary material, which is available to authorized users.

## Background

Living with a chronic rare disease often poses medical, psychological, financial and educational challenges [1]. Most rare diseases are genetic disorders, which are often debilitating and have long-term impact on the quality of life of individuals and their families [2]. Rare diseases that affect the musculoskeletal system can pose significant mobility issues from painful fractures, deformity and/or bone pain and include Osteogenesis imperfecta (OI), fibrous dysplasia (FD) and X-linked hypophosphatemia (XLH).

Historically, research in rare diseases has focused on the pathogenesis and clinical manifestations [3]. In recent years, health-related quality of life (HRQoL) has increasingly been studied. HRQoL is one’s perception and self-assessment of their physical, psychological and social domains of health [4]. Assessments of HRQoL in patients with rare diseases can be used in the evaluation of disease severity, treatments, the identification of health needs, and monitoring of progression through a patient’s life-course [5].

One reason why HRQoL in rare diseases is now of increasing interest is the change in drug development practice. In the past rare diseases were neglected by pharmaceutical companies as drug development was not commercially viable. This discrepancy was recognised worldwide and legislations now exist to encourage drug development for rare disease [6]. In the US, since 1983 when the Orphan Drug Act was passed, over 500 drugs and biological products have become available [7]. Tools to measure HRQoL can aid treatment decision-making and monitoring of treatment effects. They are required for economic evaluation and health technology assessment (HTA) of novel therapies in many countries including the UK.

HRQoL of adults with OI, XLH and FD remains poorly understood. Several previous studies [8–10] have assessed HRQoL in adults with OI using the generic instrument Short Form Health Survey (SF-36). Each study of between 15 and 30 patients showed significantly lower physical functioning compared with population norms but no difference in mental functioning scores. A similar study [11] of adults with FD again shows reduced physical function but same mental functioning compared with the US norm. In XLH, lower QoL was found compared with patients with axial spondyloarthritis [12].

However, conceptual and methodological issues have limited progress in this area. Different terminology is used to describe quality of life and widely varying evaluation tools are adopted [3]. This causes difficulty when reviewing the literature and comparing HRQoL of different disease groups. A major potential limitation of many of these studies is that they recruit participants from secondary or tertiary care settings and so findings may not be representative of adult population with rare diseases who may have milder symptoms.

The aim of this study was to describe the HRQoL of adults with OI, FD and XLH using a web-based platform that recruits participants from the community as well as hospital setting [13]. The secondary aims were to compare HRQoL between the three diseases, and perform a cost-utility simulation to calculate the maximum cost a health care system would be willing to pay for a hypothetical treatment of a rare bone disease.

## Methods

### Data sources

This study used cross-sectional data from an ongoing UK-based multi-centre prospective cohort study: RUDY (Rare and Undiagnosed Diseases Study). RUDY is a novel web-based registry and patient-driven research platform designed to improve the understanding of all aspects of rare musculoskeletal diseases [13]. Ethical approval was obtained from the UK’s South Central Research Ethics Committee (LREC 14/SC/0126).

### Study population

Potential participants were informed of the study and invited to visit the study website (www.rudystudy.org) during clinic visits at participating sites, through relevant patient groups, Facebook, Twitter and the web searches [13]. Potential participants registered on the website and provided basic demographic information. Inclusion criteria included individuals aged 18 and above with a clinical diagnosis of OI, FD or XLH. Once registered, eligible individuals were contacted via email to arrange a telephone consent appointment. Once informed consent was received, participants were granted full access to a secure personalised homepage on the study website. Demographic information collected via the study website included age, sex and patient-reported comorbidities. A recent clinic letter was requested from the participant’s doctor to verify their diagnosis. Participants were invited to complete the EQ-5D-5 L questionnaire [14] electronically via their study website homepage or using the paper version which was sent via post. Non-responders were sent reminders via email and social media during the data collection period. Participants were excluded if they had not fully completed the EQ-5D-5 L questionnaire by the final day of the data collection period. Data was collected from September 2014 to March 2016.

The EQ-5D-5 L is a widely used generic preference-based tool developed by the Euroqol group for measuring health-related quality of life [14]. The EQ-5D-5 L divides health into five dimensions (mobility, self-care, usual activity, pain/discomfort and anxiety/depression), each assessed with a 5-response-level questions [15]. Health states can then be weighted using country-specific ‘value sets’, which reflect the preferences of the general population. This results in a health utility score for each 3125 health states on a scale ranging from −0.28 (negative scores denoting health states worse than death) through 0 (dead) to 1 (full health). The recently published value set for England [16] was used in this study as a value set for the whole United Kingdom remains in development and most participants came from England.

The EQ-5D-5 L also includes a visual analogue scale (VAS) from 0 (worst health) to 100 (best health), which participants use to self-rate their health status on the day of completing the questionnaire.

### Data analysis

Descriptive statistics were used to present participants’ baseline characteristics and EQ-5D-5 L responses. Health states were converted into health utility scores using the England value set using all five response levels [16]. Mean and standard deviation of the VAS score and health utility score were calculated for each disease group. The one-way analysis of variance (ANOVA) test was used to assess statistical significant differences between mean scores for each disease group. To aid interpretation, response levels for each dimension were divided into three categories: ‘no and slight problems’ (level 1 and 2), ‘moderate; (level 3) and, ‘severe and extreme problems’ (level 4 and 5). Significant differences between the three disease groups for the proportion of participants’ responses in each category were assessed using the Fisher’s exact test. Statistical significance was set as p ≤ 0.05.

For the economic simulation, we chose OI as the case study because it reported the largest number of individuals. We split the group into tertiles to identify those individuals reporting the lowest health utility scores and therefore the greater potential improvement (Additional file 1: Figure S1). For our simulation, we considered a hypothetical treatment that would be applied to those in the lower tertile of health utility scores over a period of 10 years. Patients under treatment would attain 75% of their individual potential improvement (the difference between their reported score and 0.745, the mean of the middle tertile). We conducted sensitivity analysis on the percentage improvement producing results for 55%, 65%, 75%, 85% and 95%.

The treatment was considered to have a constant nominal cost per year. Where total costs are reported, they are discounted at the standard rate of 3.5% per year, as are health utilities according to NICE’s guidance for technology assessments [17]. Full treatment effect was assumed to be achieved linearly within the first 12 months. We further assumed no effect on mortality in either arm as the simulation was run for 10 years only. Health utility was however decreased every year by the coefficient found associated with age for all OI patients (−0.005 per year) in a linear regression on reported health utility. Quality-adjusted life years were estimated for each individual in each arm and added over the 10 year period.

All data analysis was carried out using STATA IC version 14.

## Results

### Baseline characteristics

A total of 174 participants were recruited to the study. 109 participants fully completed the EQ-5D-5 L questionnaire (response rate 63%). The baseline characteristics of respondents are shown in Table 1 and there were no statistically significant difference between baseline characteristics of the responders and non-responders (p > 0.05) for age, gender and number of comorbidities. Amongst those diagnosed with OI and using Sillence’s classification, 18 participants had type I, five had type III, five had type IV and 15 reported an unknown type.

**Table 1:** Descriptive statistics of respondents

	Diagnosis			
	OI	FD	XLH	Total
*n*	43	42	24	109
Age (mean (years) (SD))	40.4 (14.4)	44.3 (14.5)	46.3 (16.3)	43.2 (14.9)
Age (range years)	18–70	18–75	22–78	18–78
Gender n (%) Female	33 (77)	29 (69)	19 (79)	81 (74)

### EQ-5D-5 L response levels

**Fig. 1:**
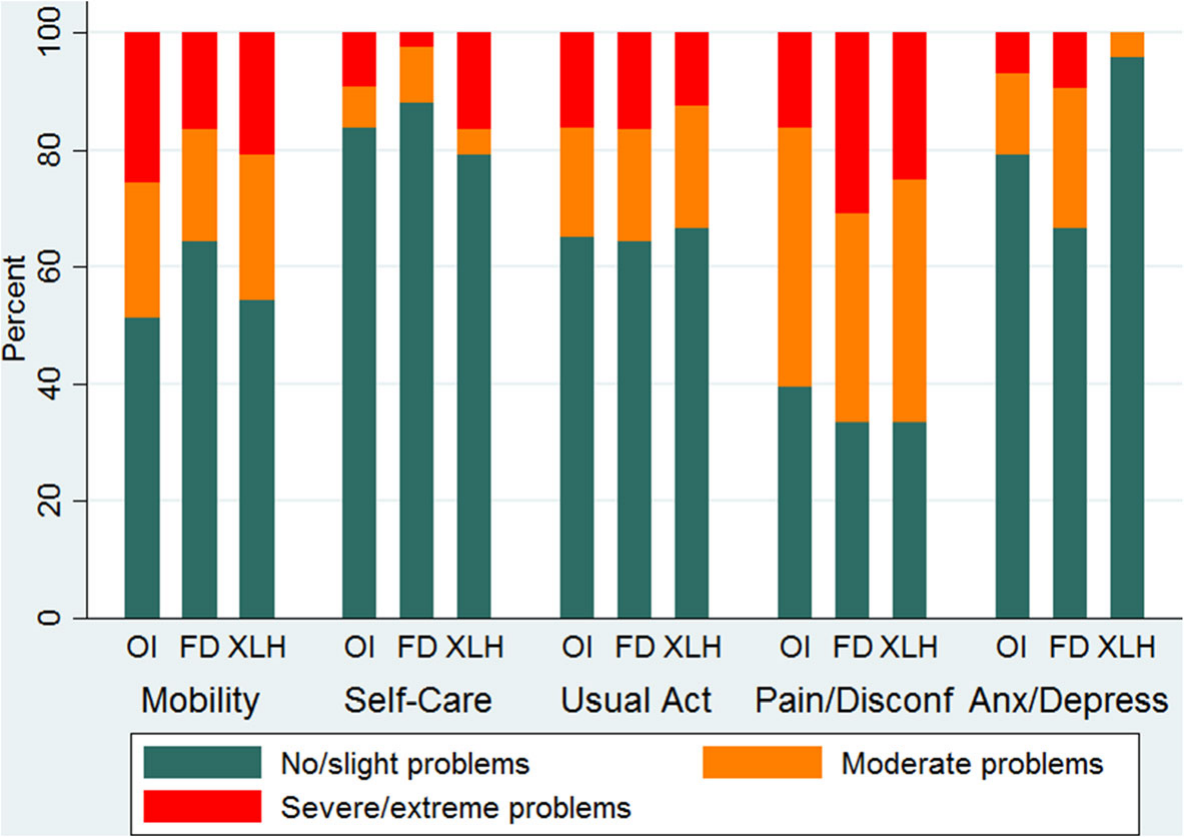
Levels of problems by EQ-5D-5 L dimension for patients in each disease group

Response level frequencies for each domain by disease is shown in Table 2. The response groups by no/slight vs. moderate vs. severe/extreme are shown in Fig. 1. Pain/discomfort was the most problematic domain for participants within all three diseases. Overall 94% of respondents reported problems with pain or discomfort. Thirty one percent of adults with FD reported severe/extreme problems with pain, with a quarter of those with XLH and 16% of those with OI reporting similar levels of problem.

**Table 2:** Percentage of participants reporting level 1–5 for each EQ-5D-5 L dimension by disease group

EQ-5D-5 L dimensions	Distribution of responses n (%)			
	Diagnosis			
	OI	FD	XLH	Total
Mobility				
No problems	8 (19)	18 (43)	3 (13)	29 (27)
Slight problems	14 (33)	9 (21)	10 (42)	33 (30)
Moderate problems	10 (23)	8 (19)	6 (25)	24 (22)
Severe problems	6 (14)	7 (17)	2 (8)	15 (14)
Extreme problems	5 (12)	0 (0)	3 (13)	8 (7)
Self-care				
No problems	26 (61)	26 (62)	12 (50)	64 (59)
Slight problems	10 (23)	11 (26)	7 (29.)	28 (26)
Moderate problems	3 (7)	4 (10)	1 (4)	8 (7)
Severe problems	2 (5)	1 (3)	2 (8)	5 (5)
Extreme problems	2 (5)	0 (0)	2 (8)	4 (4)
Usual activity				
No problems	15 (35)	14 (33)	6 (25)	35 (32)
Slight problems	13 (30)	13 (31)	10 (42)	36 (33)
Moderate problems	8 (19)	8 (19)	5 (21)	21 (19)
Severe problems	5 (12)	4 (10)	3 (13)	12 (11)
Extreme problems	2 (5)	3 (7)	0 (0)	5 (5)
Pain/Discomfort				
No problems	3 (7)	1 (2)	2 (8)	6 (6)
Slight problems	14 (33)	13 (31)	6 (25)	33 (30)
Moderate problems	19 (44)	15 (36)	10 (42)	44 (40)
Severe problems	4 (9)	9 (21)	5 (21)	18 (17)
Extreme problems	3 (7)	4 (10)	1 (4)	8 (7)
Anxiety/Depression				
No problems	17 (40)	16 (38)	10 (42)	43 (39)
Slight problems	17 (40)	12 (29)	13 (54)	42 (39)
Moderate problems	6 (14)	10 (24)	1 (4)	17 (16)
Severe problems	3 (7)	4 (10)	0 (0)	7 (6)
Extreme problems	0 (0	0 (0)	0 (0)	0 (0)

Mobility also caused problems to a significant proportion of respondents. Overall 73% reported some level of problem with mobility and severe/extreme problems were reported by 21% of total respondents. FD respondents compared with the other condition groups reported mobility as least problematic, 43% reporting no problems compared with 19% in the OI group and 13% in the XLH group.

Self-care was least problematic overall for participants (82% reported no or slight problems). Less than 10% of all respondents reported severe or extreme problems with self-care.

Overall the majority of respondents (68%) reported problems with usual activity to varying extent. However most reported slight problems (30% OI, 31% FD, 42% XLH) and the percentage of respondents reporting problems decreased as the severity of the response option increased.

Low levels of severe or extreme anxiety and depression were reported in all three groups (less than 10% in each group). Overall most respondents reported no and slight problems in this domain (78%). The highest percentage of FD respondents reported moderate problems (24%) compared to the other groups.

There was no statistically significant difference, using the Fisher’s exact test, in responses between disease groups for all dimensions of the EQ-5 L-5D (Mobility *p* = 0.775, Self-care *p* = 0.322, Usual activities *p* = 1.000, Pain/discomfort *p* = 0.627, Anxiety/depression *p* = 0.100). Among OI respondents linear regression showed a statistically significant positive correlation between age and response level (*p* = 0.01, Pearson’s *r* =0.39) in the Usual Activities domain, meaning that older age is associated to more problems in performing daily activities. For all other domains no significant correlation between age and response levels was found across all three disease groups.

### Health utility score

**Fig. 2:**
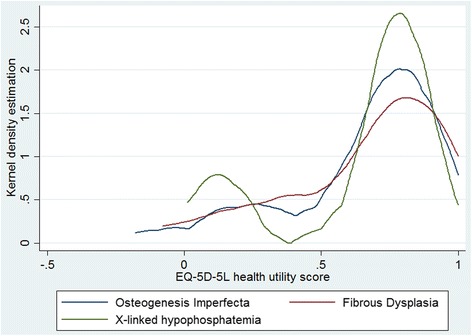
Distribution of EQ-5D-5 L health state summary score by disease group

The mean health utility scores generated using the England value set [16] were high for all disease groups (OI 0.656, SD 0.283; FD 0.656, SD 0.288; XLH 0.648, SD 0.290; Overall 0.654, SD 0.284), with wide distributions (Fig. 2). For all three conditions the distribution of health utility was bimodal, this pattern was most evident for XLH. No statistically significant difference between the three groups for the health utility score was found (*p* = 0.993).

### VAS score

**Fig. 3:**
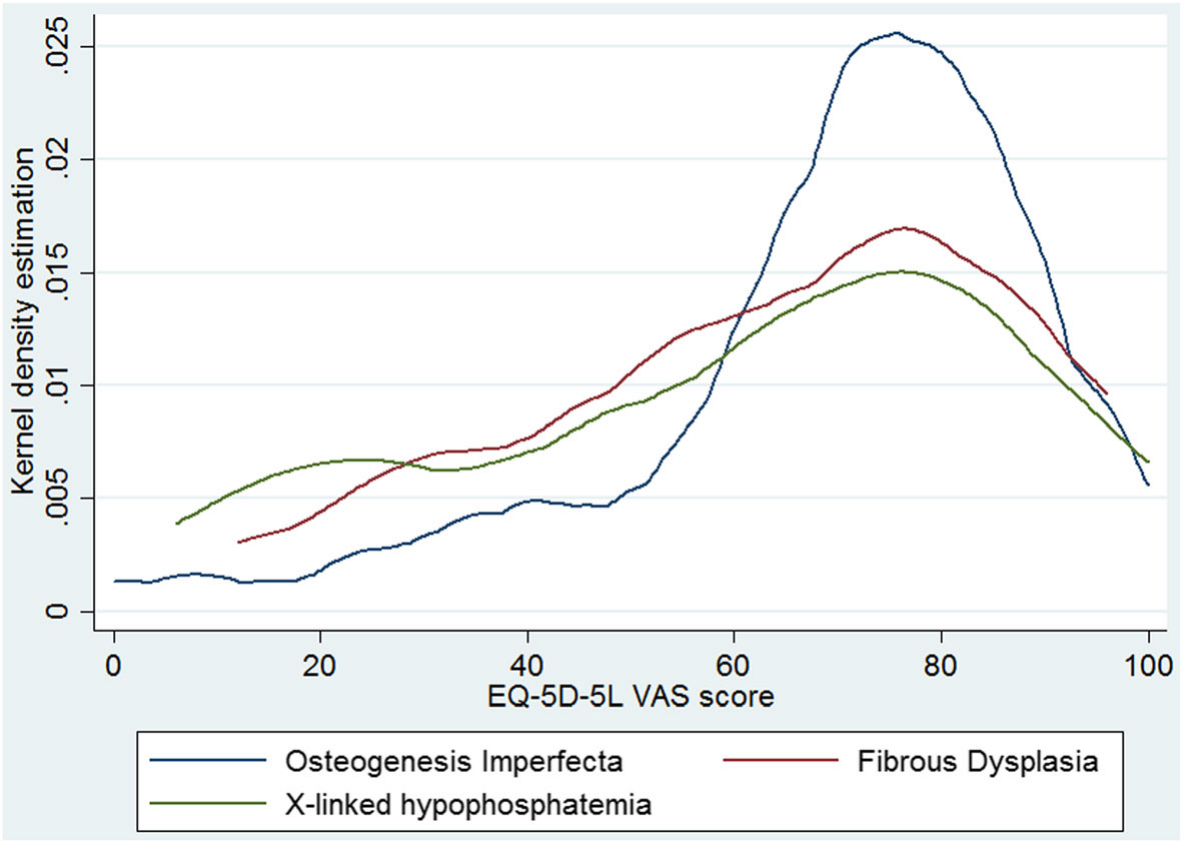
Distribution of EQ-5D-5 L Visual Analogue Score by disease group

The reported VAS scores were 69.4 (SD 21.4) for OI, 64.1 (SD 23.0) for FD and 60.8 (SD 26.9) for XLH adult patients. For all diseases the distribution was skewed to high VAS scores (Fig. 3). The graph shows a higher proportion of respondents with OI reporting high VAS scores compared with the other diseases. A statistically significant negative correlation between VAS score and age was found in the OI group using linear regression (*p* = 0.005 Pearson’s *r* = −0.42), showing that older age is associated with worse perception of self-rated health.

### Economic simulation

**Fig. 4:**
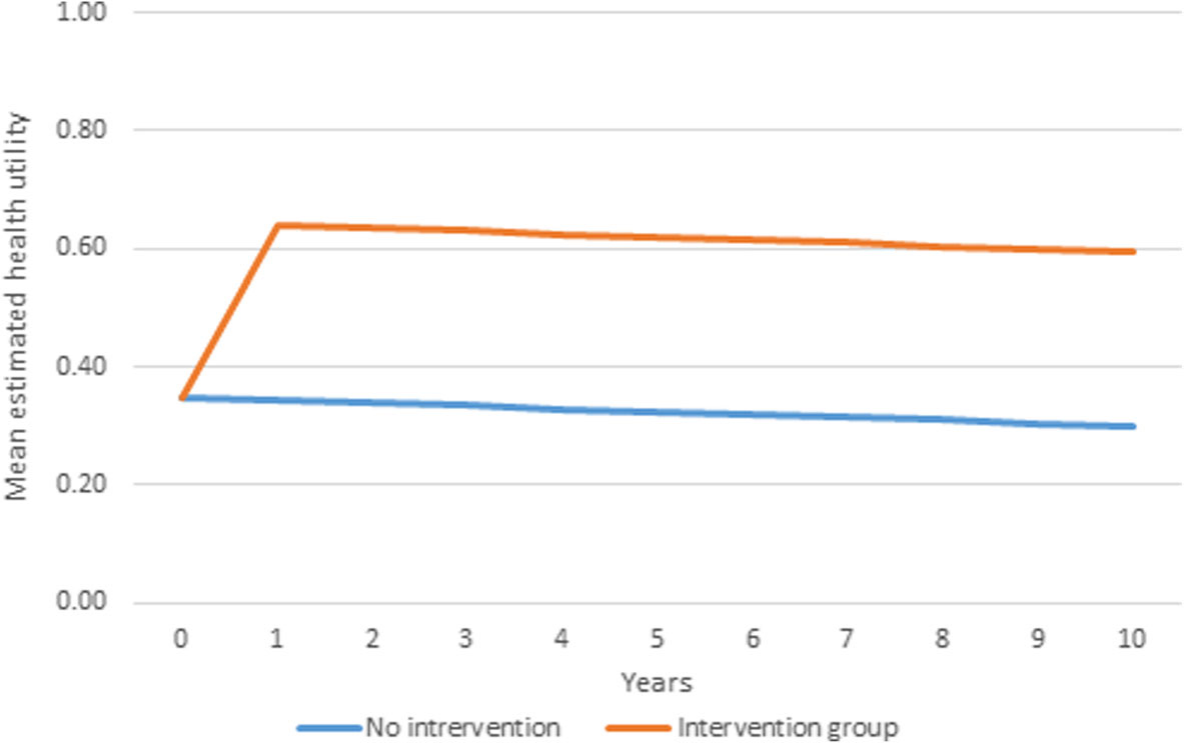
Projected health utility estimate by treatment group

For our base case, where 75% of the individual potential improvement would be achieved during the first 12 months, the simulated treatment group would have accumulated 78 QALYs over the following 10 years (mean of 5.2 per person). The no-treatment group would have accrued 41 QALYs in total (2.8 per person), leaving the simulated treatment with an associated impact of +2.5 QALYs gained per person during the 10-year period. Given the small sample size, the mean QALY gain reported a high standard deviation (1.66), thus producing a wide confidence interval (−0.78 to 5.72). Figure 4 shows the progression over time of the simulated mean health utility index for each group. The QALY gain is related to the individual potential improvement at one year. Expected QALY gain per person over a 10 year period for other potential improvements were +1.83 (55% improvement during the first year), +2.15 (65%), +2.79 (85%) and +3.11 (95%).

Based on these results, we calculated the corresponding maximum annual discounted cost that a health care system would be willing to pay for the treatment at various thresholds of cost-effectiveness over the 10 years (Table 3).

**Table 3:** Maximum annual cost of intervention (over 10 years) per patient to be cost-effective at various cost-effectiveness thresholds

Willing-to-pay threshold	% of potential improvement attained during first 12 months of treatment				
	55%	65%	75% (Base case)	85%	95%
20,000	4,254	5,000	5,742	6,481	7,218
30,000	6,382	7,499	8,613	9,722	10,827
40,000	8,509	9,999	11,484	12,963	14,436
50,000	10,636	12,499	14,355	16,203	18,045
60,000	12,763	14,999	17,226	19,444	21,653
70,000	14,890	17,498	20,097	22,685	25,262
80,000	17,017	19,998	22,967	25,925	28,871
90,000	19,145	22,498	25,838	29,166	32,480
100,000	21,272	24,998	28,709	32,406	36,089

For example, a health care system willing to pay up to £50,000 for each additional QALY gained by an intervention for OI patients, would be willing to pay £14,355 annually for such treatment achieving 75% of the potential improvement during the first year. After the appropriate discounting has been applied, this annual cost amounts to £123,561 over the 10 years of treatment which, expected to improve patient outcomes by an average 2.47 QALYs over the period, would equate to an incremental cost-effectiveness ratio of £50,000 per QALY. Higher treatment costs would not be considered value for money for a health care system with this willingness to pay, whereas a lower cost would be likely to represent value for money for this health care system.

## Discussion

This is the first study to quantitatively measure and compare the HRQoL of adults with OI, FD and XLH. We found on average the recruited participants with the three diseases have a high HRQoL, as shown by high mean health utility scores and correspondingly high mean VAS scores. There was no significant difference in distribution of EQ-5D-5 L responses between the three rare disease groups.

As the England value set for EQ-5D-5 L response weighting has only recently been published [16], there are currently no studies of other disease groups or the general population to directly compare the health utility scores with. However, the VAS score, which does not require weighting to interpret, has been reported for other disease groups. A multi-country study of 3919 patients with a range of medical conditions reports a mean VAS score of 60 (SD 25) for adults with rheumatoid arthritis and 52 (SD 19) for those with chronic back pain [18]. Non-institutionalised adults with cystic fibrosis reported a mean VAS score of 62 (SD 20) [19]. The mean scores for OI (67.6 SD 22.7), FD (64.4 SD 26.1) and XLH (63.3 SD 25.2) were marginally higher than these conditions.

When analysing the individual domains of the EQ-5D-5 L we found that for all three disease groups pain/discomfort was the most problematic domain followed by mobility, suggesting these aspects of life should be prioritized for health care research and interventions. Despite physical burdens, the majority of participants reported low levels of anxiety/depression, and minimal problems with self-care and usual activity across all three conditions. This could indicate the ability of adults with OI, XLH and FD to cope with the physical limitations of their chronic disease and prevent these from impinging on their daily life and mental health. Previous studies [8, 11, 20] of adults with FD and OI have also reported high life satisfaction and ‘resilience’.

One study has reported the distribution of EQ-5D-5 L responses of 996 adults from the general population in England [21]. The age range of the general population sample was similar to our cohort and 26.9% had a long lasting illness. Overall the respondents with OI, FD and XLH reported more problems in every domain compared to the general population. Twenty-six percent of the general population sample reported any problems (level 2 and above) with mobility compared with 81% of OI, 56% of FD and 83% of XLH respondents. Likewise 41.6% of the general population reported pain and discomfort problems in comparison with 94% overall across the three disease groups. Though self-care was the domain that caused least problems for our respondents, the percentage reporting any problems (overall 41%) was still much higher than in the general population sample (9.2%). This suggests that although this study reports overall high HRQoL in OI, FD and XLH, adults do have more problems in health-related aspects of their life, compared with the general population, for which necessary support should be available.

The overall high utility scores for participants with OI, FD and XLH, and the fact that these were higher than VAS scores for some participants suggest the health utility estimated through the EQ-5D-5 L instrument may be over-estimating HRQoL and failing to capture the negative effects of these rare chronic conditions. This has been shown for other physical and psychological chronic conditions [22]. When health utility is then used for resource allocation for these conditions underfunding would be likely. This is therefore an argument for disease specific HRQoL measures, which would be more sensitive in detecting changes in HRQoL for a particular condition. Such tools are currently being developed for children with OI [23] and we suggest similar instruments would be useful for adults with rare bone diseases.

Nonetheless we were able to use the EQ-5D-5 L utility scores of OI participants to generate a cost-utility simulation, which has not previously been done. This gives industries and health-care providers a target cost figure for the development of treatments and services for adults with OI based on various cost-effectiveness thresholds. However, undoubtedly, given that rare disease individuals face an adverse scarcity of available treatments, any decision of reimbursement of an intervention should consider, in addition to its value for money, other ethical issues of inequality in access to adequate health care.

We acknowledge the limitations in the study methodology. The narrow range of baseline characteristics that were collected from participants limits the findings of this study. Factors such as disease severity, previous treatments, occupation, marriage status and education may have confounding effects on HRQoL.

Other potential sources of bias were that most participants were female and participants were self-referred via the Internet, which may indicate a better health state or bias towards younger computer literate population who may have fewer mobility/self-care problems. However, the large heterogeneity in results suggests that the participants were not only those with better health and confirms the RUDY platform captures a wide spectrum of disease severity. The wide range of health utility and VAS scores reported also shows the high variability in how OI, FD and XLH impact on individuals’ HRQoL. It may be important to assess patient’s self-reported HRQoL alongside objective physical measures to determine disease severity. Tosi et al. [20] also suggested this in a study of adults with OI. They found self-reported symptom severity to not always correlate with disease severity classified using height and Sillence’s types.

For OI participants varying disease severity was expected, as participants with different types of OI were included. However, 34% of respondents with OI had an unknown type and analysing the relationship between HRQoL Sillence’s types was not possible due to the small sample size. In the OI group there was statistically significant correlation between age and decreasing VAS score as well as age and increasing problems with usual activity. This suggests reduced self-perceived quality of life with age might be related to worsening health, which restricts their usual activities.

The EQ-5D-5 L is a generic health-related quality of life measure and may not capture specific problems for this patient group; also the utility score is derived from the preferences of the general population and the preferences for patients with rare bone diseases may differ, giving different utility weights.

The economic simulation is also limited by a small sample size. As the analysis was conducted for 15 patients only, this simulation is expectedly associated to a significant level of uncertainty, confirmed by a wide 95% confidence interval over the QALY gain per person. Nevertheless, this simulation does not intend to provide hard estimates contextualized by the uncertainty reflected in confidence intervals, but rather to examine possible scenarios of treatment costs based on expected levels of health gains under the assumptions made and based on unique health utility scores reported by a small group of individuals with rare musculoskeletal diseases. Further replication studies are needed to validate these findings in larger numbers.

## Conclusion

In conclusion, we compared and found similar HRQoL of adults with OI, FD *and* XLH. Our findings show that overall, despite the burdens of their rare chronic conditions, adults with OI, FD and XLH maintain a good quality of life. For these patients, pain/discomfort is the aspect of life future treatment developments should aim to improve. Our findings suggest disease-specific HRQoL measures for these conditions would be beneficial and an area for future research. Finally we have, for the first time, conducted a simulation to examine the maximum cost that a health care system would be willing to pay for a potential treatment for OI based on various cost-effectiveness thresholds.

## Additional file

Additional file 1: Figure S1. Health utility by EQ-5D tertiles for adults with Osteogenesis Imperfecta. The top tertile scored between 0.817 and 1 and averaged 0.896, and those in the middle tertile reported scores ranging from 0.676 to 0.816, with a mean estimated health utility of 0.745. The lowest tertile comprised individuals scoring as low as −0.180 and no higher than 0.673, with a mean for the group at 0.350. (TIF 1547 kb)

## Abbreviations

ANOVA: One-way analysis of variance
FD: Fibrous dysplasia
HRQoL: Health-related quality of life
HTA: Health technology assessment
OI: Osteogenesis imperfecta
QALY: Quality-adjusted life year
VAS: Visual analogue scale
XLH: X-linked hypophosphatemia
