# Supplementary figures and images for: Health-related quality of life and a cost-utility simulation of adults in the UK with osteogenesis imperfecta, X-linked hypophosphatemia and fibrous dysplasia
Source: Orphanet J Rare Dis. 2016 Nov 28;11:160. doi: 10.1186/s13023-016-0538-4 (PMC5126812; doi:10.1186/s13023-016-0538-4)

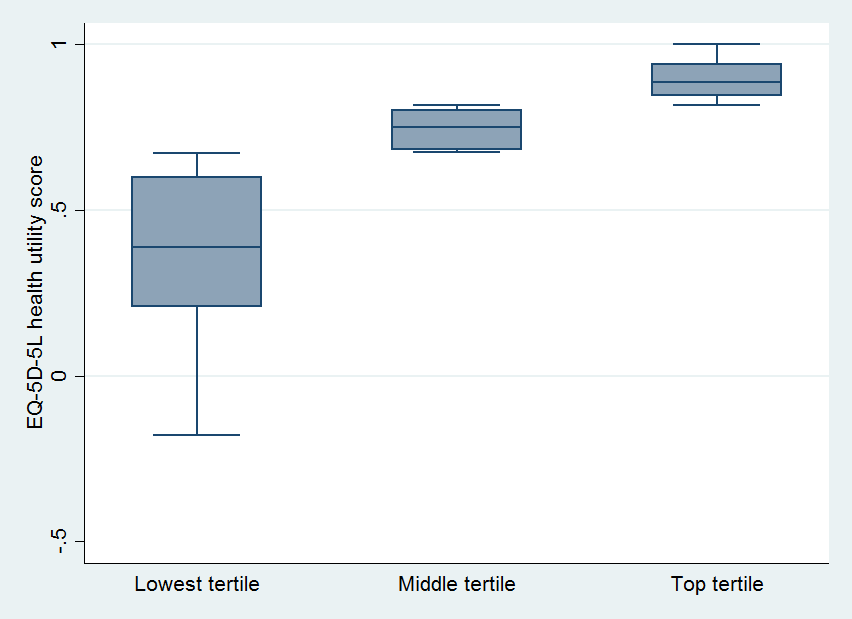

Supplement: Additional file 1: Figure S1. — Health utility by EQ-5D tertiles for adults with Osteogenesis Imperfecta. The top tertile scored between 0.817 and 1 and averaged 0.896, and those in the middle tertile reported scores ranging from 0.676 to 0.816, with a mean estimated health utility of 0.745. The lowest tertile comprised individuals scoring as low as −0.180 and no higher than 0.673, with a mean for the group at 0.350. (TIF 1547 kb) [file 13023_2016_538_MOESM1_ESM.tif]
